# Obstructive sleep apnea and long COVID syndrome: a scoping review of current evidence in adults

**DOI:** 10.3389/frsle.2026.1990528

**Published:** 2026-10-29

**Authors:** Munyra I. Alhotye, Tareq F. Alotaibi, Manar M. Alotaibi, Renad M. Alojyri, Alhanouf G. Almutairi, Hala A. Almutairi, Renad S. Alghuraibi, Afaf M. Alrashdi, Arwa H. Alruwaili, Mobarak K. Alqahtani

**Affiliations:** 1 Department of Respiratory Therapy, College of Applied Medical Sciences, King Saud Bin Abdulaziz University for Health Sciences, Riyadh, Saudi Arabia; 2 King Abdullah International Medical Research Center, Riyadh, Saudi Arabia; 3 National Guard Health Affairs, Riyadh, Saudi Arabia; 4 Department of Rehabilitation Sciences, College of Health and Rehabilitation Sciences, Princess Nourah Bint Abdulrahman University, Riyadh, Saudi Arabia

**Keywords:** COVID-19, long covid, obstructive sleep apnea, pathophysiology, post-acute sequelae of SARS-CoV-2 infection

## Abstract

**Background:**

Obstructive Sleep Apnea (OSA) is a prevalent sleep disorder characterized by intermittent hypoxia and sleep fragmentation, contributing to systemic inflammation and autonomic dysfunction. Long COVID refers to persistent symptoms following acute COVID-19 infection. Emerging evidence suggests an association between OSA and persistent post-COVID-19 symptoms, potentially mediated by overlapping biological pathways; however, the direction and causality of this relationship remain uncertain.

**Aim:**

This scoping review aimed to examine the current evidence regarding the different associations between OSA and Long COVID in adults, summarize the reported clinical findings, and explore the proposed pathophysiological mechanisms linking OSA and Long COVID.

**Methods:**

Following PRISMA-ScR guidelines, PubMed, Embase, Scopus, Web of Science, Cochrane Library, Google Scholar, and ClinicalTrials.gov were searched for studies published between 2020 and 2026. Eligible studies included observational studies, clinical trials, and reviews involving adults with OSAand/or Long COVID syndrome. Two independent reviewers screened studies, extracted data, and synthesized findings thematically.

**Results:**

Of 184 identified records, 11 studies met the inclusion criteria. Evidence suggested different associations between OSA and Long COVID across the included studies. Proposed mechanisms included intermittent hypoxia, systemic inflammation, autonomic dysregulation, sleep fragmentation, and circadian disturbance; however, these mechanisms were not consistently measured across studies. Reported Long COVID manifestations among patients with OSA included fatigue, respiratory impairment, cognitive dysfunction, and cardiovascular complications. Evidence regarding the impact of OSA treatment on Long COVID outcomes was limited.

**Conclusions:**

Current evidence suggests an association between OSA and Long COVID, although the nature and direction of this association vary across studies. However, the evidence remains limited and heterogeneous. Further longitudinal studies are needed to clarify this relationship and determine whether effective OSA management can improve Long COVID outcomes.

## 1 Introduction

Coronavirus Disease 2019 (COVID-19) has both short-term and long-term health effects worldwide (Labarca et al., 2022). While many patients recover completely, a significant portion continue to experience symptoms after the acute phase of the illness. Long COVID syndrome, as defined by the World Health Organization, is a condition occurring in individuals with a history of probable or confirmed SARS-CoV-2 infection, usually 3 months from onset, with symptoms lasting at least 2 months and not explained by an alternative diagnosis (Soriano et al., 2022). The National Academies of Sciences, Engineering, and Medicine (NASEM) define Long COVID as an infection-associated chronic condition that occurs following SARS-CoV-2 infection and persists for at least 3 months. It may present as a continuous, relapsing and remitting, or progressive disease state affecting one or more organ systems (National Academies of Sciences Engineering Medicine, 2024; Scholkmann and May, 2023; Suzana et al., 2022; O'Keefe, 2023). A pooled worldwide prevalence estimates from a recent systematic review and meta-analysis suggested that over 40% of individuals recovering from COVID-19 experience Long COVID symptoms (Sk Abd Razak et al., 2024). These symptoms can range from fatigue and shortness of breath to cough, chest pain, memory or concentration problems, and sleep disorders. Long COVID can affect anyone, regardless of the severity of the initial infection, including individuals with mild or no symptoms. It is now recognized as a distinct clinical entity due to its profound impact on patients' daily functioning and quality of life (Geng et al., 2022; Healey et al., 2022).

Obstructive Sleep Apnea (OSA) is a common sleep disorder characterized by repeated episodes of low oxygen levels (intermittent hypoxia) and disrupted sleep patterns (sleep fragmentation), affecting an estimated 936 million adults worldwide (Benjafield et al., 2019). OSA is diagnosed and classified according to the apnea–hypopnea index (AHI), which represents the number of apneas and hypopneas per hour of sleep. OSA is classified as mild (AHI 5–15 events/h), moderate (AHI 15–30 events/h), or severe (AHI >30 events/h) [American Academy of Sleep Medicine Task Force, 1999]. It has been linked to poorer outcomes in patients recovering from COVID-19 (Popovici et al., 2024; Park et al., 2011; Wali et al., 2015). Patients with OSA may be more likely to develop COVID-19, possibly because sleep disturbances such as insomnia and reduced sleep quality can weaken the immune system and increase vulnerability to infections (Zhou et al., 2024).

In addition, individuals with pre-existing OSA appear to be more likely to experience persistent symptoms after COVID-19 compared to the general population. A recent study found that 25% of patients with OSA had impaired lung diffusion capacity (DLCO) 1 year after infection, while only 3.6% of non-OSA patients showed similar findings (Quan et al., 2024). These findings suggest that OSA may not only increase the risk of COVID-19 complications, but may also contribute to the development and severity of Long COVID syndrome.

Preexisting obstructive sleep apnea (OSA) is markedly associated with an increased risk of probable Post-Acute Sequelae of SARS-CoV-2 (PASC) among adults (Mandel et al., 2023; Cinthya Pena-Orbea, 2023). This elevated risk for PASC persists even after adjusting for vital confounding factors such as obesity, acute infection severity, and preexisting comorbidities (Mandel et al., 2023). Biologically, it is hypothesized that OSA-related mechanisms specifically intermittent hypoxia, sleep fragmentation, and systemic inflammation create a microenvironment that may facilitate the development of post-viral sequelae (Cinthya Pena-Orbea, 2023).

Although research on the association between OSA and poor COVID-19 outcomes is emerging, there is still a gap in understanding the role of OSA in the development and persistence of Long COVID syndrome (Geng et al., 2022). While many studies have examined each condition separately, few have explored their interaction, particularly regarding shared biological mechanisms such as chronic inflammation, autonomic nervous system dysfunction, and intermittent oxygen deprivation (Geng et al., 2022; Luo et al., 2024). In addition, there is limited clinical evidence on whether OSA worsens Long COVID syndrome or whether treatments such as CPAP therapy may support recovery (Geng et al., 2022).

To better understand the association between OSA and Long COVID syndrome and identify at-risk populations, this review was guided by the primary research question:

What is the current evidence regarding the association between obstructive sleep apnea and Long COVID in adults? To address this, we aimed to examine the different associations reported between OSA and Long COVID, summarize the clinical findings reported across the included studies, and explore the potential pathophysiological mechanisms linking OSA and Long COVID.

## 2 Methods

This scoping review was reported in accordance with the PRISMA-ScR guidelines. The review aimed to explore the association between obstructive sleep apnea (OSA) and Long COVID syndrome, including potential mechanisms, clinical outcomes, and treatment-related findings. Additionally, this scoping review has been registered on the Open Science Framework (https://doi.org/10.17605/OSF.IO/265HA).

### 2.1 Eligibility

Studies were selected for inclusion based on the Population, Concept, and Context (PCC) framework. Briefly, this review considered studies involving adults (≥18 years old) diagnosed with obstructive sleep apnea (OSA) and/or Long COVID syndrome, including relevant post-COVID and COVID-19-related outcomes, published between 2020 and 2026. A comprehensive and detailed description of the predefined inclusion and exclusion criteria is provided in Table 1.

**Table 1:** PCC framework and eligibility criteria used for study selection.

PCC element	Description
Population	Adults (≥18 years old) diagnosed with obstructive sleep apnea (OSA) and/or Long COVID syndrome.
Concept	The relationship between OSA and Long COVID syndrome, including potential mechanisms such as intermittent hypoxia, systemic inflammation, autonomic dysfunction, cardiovascular effects, endothelial dysfunction, coagulation abnormalities, metabolic disturbances, and insulin resistance. Studies examining OSA within Long COVID populations, related post-COVID outcomes, or the broader OSA–COVID-19 relationship, including the impact of OSA treatment (e.g., CPAP and lifestyle modifications) were also considered.
Context	Studies published between 2020 and 2026 investigating the relationship between OSA and Long COVID syndrome, including post-COVID outcomes, sleep-related manifestations, potential mechanisms, and related COVID-19 outcomes in adults.
Study design	Randomized controlled trials (RCTs), non-randomized controlled trials (nRCTs), cohort studies, case-control studies, cross-sectional studies, systematic reviews, meta-analyses, and qualitative studies.
Other criteria	English-language studies with full-text availability.
Exclusion criteria	Pediatric populations (< 18 years old). For studies including both adults and pediatric participants, only adult data will be considered where separately available; sleep disorders other than OSA (e.g., insomnia and narcolepsy), case reports, editorials, letters, and expert opinions, non-peer-reviewed preprints, animal studies, studies not investigating an association relevant to OSA and Long COVID or COVID-19-related outcomes, non-English articles or studies without full-text availability.

### 2.2 Search strategy

PubMed, Embase, Scopus, Web of Science, Cochrane Library, Google Scholar, and ClinicalTrials.gov were searched for studies published between 2020 and 2026. Search terms included combinations of “obstructive sleep apnea,” “OSA,” “Long COVID,” “PASC,” “hypoxia,” “inflammation,” and “autonomic dysfunction.” Controlled vocabulary and MeSH terms were used where applicable. The full search strategy for each database is provided in Table 2. Google Scholar results were sorted by relevance and limited to the publication years 2020–2026. The final search was conducted on August 28, 2026.

**Table 2:** Full search strategies across databases.

Database	Search query (Copy & Paste in Search Bar)	Filters to apply
PubMed	(“Obstructive Sleep Apnea” OR “OSA” OR	Language: English
	“Sleep-Disordered Breathing”) AND (“Long	Publication Date: 2020–2026
	COVID” OR “Post-COVID Syndrome” OR	
	“PASC”) AND (“Hypoxia”OR “Inflammation” OR “Autonomic Dysfunction”) AND (“Dyspnea” OR “Fatigue” OR “Cognitive Impairment”)	Study Type: RCTs, Cohort Studies, Systematic Reviews
Scopus	TITLE-ABS-KEY (“Obstructive Sleep Apnea” OR “OSA”) AND (“Long COVID” OR “Post-COVID Syndrome”) AND (“Hypoxia” OR “Inflammation”)	Same as PubMed
Web of science	TS=(“Obstructive Sleep Apnea” OR “OSA”) AND TS=(“Long COVID” OR “Post-COVID Syndrome”) AND TS=(“Hypoxia” OR “Inflammation”)	Same as PubMed
Cochrane library	“Obstructive Sleep Apnea” AND “Long COVID” AND “Hypoxia”	Same as PubMed
Embase	(‘obstructive sleep apnea'/exp OR ‘OSA') AND (‘long covid'/exp OR ‘post-covid syndrome') AND (‘hypoxia'/exp OR ‘inflammation')	Same as PubMed
Google scholar	“Obstructive Sleep Apnea” AND “Long COVID” AND (“Hypoxia” OR “Inflammation”) site:ncbi.nlm.nih.gov OR site:nih.gov OR site:who.int	Sort by: Relevance
		Year Range: 2020–2026
clinicaltrials.gov	“Obstructive Sleep Apnea” AND “Long COVID”	Recruitment Status: Completed & Ongoing
		Study Type: Interventional & Observational

### 2.3 Study selection

Search results were imported into EndNote 9.1 (Clarivate Analytics), and duplicate records were removed. Records were subsequently screened using Rayyan. Two independent reviewers screened titles, abstracts, and full-text articles according to predefined eligibility criteria. Disagreements were resolved through discussion, and a third reviewer was consulted when necessary. Reasons for exclusion during full- text screening were documented in the PRISMA flow diagram.

### 2.4 Data extraction and synthesis

Data were charted using a standardized Excel form. Charted information included study characteristics, participant demographics, study design, OSA- related findings, Long COVID manifestations, clinical outcomes, and proposed mechanisms. For each included study, the reported Long COVID /PASC definition and symptoms-duration/time-frame criterion were also extracted and presented in Tables 3a, b, 4a, b; when not reported, this was recorded as Not reported and no definition was inferred. Findings were synthesized thematically to identify common patterns, clinical outcomes, and research gaps. Study heterogeneity was considered during interpretation by documenting variations in study populations, diagnostic criteria, outcome measures, and study settings.

**Table 3a:** Study characteristics.

Study	Country/design	Sample/population	Age/gender	OSA definition/severity
Jarosch et al. (2024)	Country: Germany Study Design: Prospective cross sectional study	Sample Size: 30 Population: Post COVID-19 Syndrome Group (n = 20): Individuals with post-COVID-19 syndrome recruited during inpatient pulmonary rehabilitation. Control Group (n = 10): Healthy volunteers without a history of SARS-CoV-2 infection.	Mean Age: PCS: 50 ± 11 years; Controls: 46 ± 10 years Gender (%): NR	OSA Definition: OSA was defined based on Apnea–Hypopnea Index (AHI) measured by overnight polysomnography (PSG) OSA Severity: Mild (5–14), Moderate (15–29), Severe (≥30 events/hour)
Quan et al. (2024)	Country: USA Study Design: Cross-sectional	Sample Size: 24803 Population: General population sample of US adults (N = 24,803). COVID-negative (n = 14,479) COVID-positive (n = 10,324) 41.6%	Mean Age: COVID-negative: 50.6 ± 18.0 years COVID-positive: 40.9 ± 16.1 years Gender (%): COVID-negative: 48.8% male, 51.2% female; COVID-positive: 49.1% male, 50.9% female.	OSA Definition: OSA defined as self-reported current diagnosis or symptom-based criteria (snoring + witnessed apnea/sleepiness). OSA Severity: Not assessed.
Labarca et al. (2022)	Country: Chile Study Design: Observational Prospective cohort study	Sample Size: 60 (57 at final follow-up) Population: Adults with confirmed COVID-19 infection.	Mean Age: 46.4 years- SD _+13.1 Gender (%): Male: 53.3%	OSA Definition: Respiratory disturbance index (RDI) ≥5 events/hour OSA Severity: RDI ≥15 events/hour
Coelho et al. (2024)	Country: USA Study Design: Cohort study	Sample Size: 42 Population: Adults with confirmed COVID-19 diagnosis	Mean Age: 50.95 ± 13.48 years Gender (%): Male 13/42 (29%) - Female 29/42 (71%)	OSA Definition: Diagnosed based on ICSD-3 criteria in an AASM-accredited sleep center OSA Severity: Not reported
Zhou et al. (2024)	Country: Multi-country Study Design: meta-analysis study	Sample Size: 8664026 Population: Adults with pre-existing sleep disturbances from included observational studies	Mean Age: Varied across included studies Gender (%): Mixed male and female	OSA Definition: Based on definitions used in individual included OSA Severity: Not reported
Mandel et al. (2023)	Country: USA Study Design: EHR based analysis	Sample Size: N3C = 1,783,940/PCORnet = 333,642/PEDSnet = 106,262 adults with SARS-CoV-2 infection Population: Adults with confirmed SARS-CoV-2 infection in RECOVER EHR database comparison between patients with pre-existing OSA and those without OSA. Patient N3C and PCORnet restricted to adults aged 21 and older, while PEDSnet was restricted to children under age 21	Mean Age: NR Gender (%): N3C-Male: 38.03%/Female: 61.94% PCORnet-Male: 39.91%/Female: 60.06% PEDSnet-Male: 51.46%/Female: 48.54%	OSA Definition: Pre-existing documented OSA diagnosis in EHR prior to COVID-19 infection (ICD codes based) OSA Severity: Not reported
Ljunggren et al. (2026)	Country: Sweden Study Design: Population-based, nationwide Cohort study using multi-register data	Sample Size: 1,932,081 COVID-19 infected individuals; 11,407 had registered OSA Population: Adults ≥18 years in Sweden with registered COVID-19 infection from January 2020–May 2022	Mean Age: OSA group: 54.0 years (median, IQR 46–62); overall COVID population: 41.0 years Gender (%): OSA group Male: 70.4%	OSA Definition: Registered OSA based on the Swedish National Register of Respiratory Failure (Swedevox), specifically patients who started PAP treatment for OSA during 2015–2019 OSA Severity: AHI: mild < 15, moderate 15– < 30, severe ≥30 events/h. ODI categorized using the same cutoffs. Median AHI = 30.0 events/h; median ODI = 26.0 events/h
Mery et al. (2026)	Country: Spain Study Design: Observational, descriptive, prospective study	Sample Size: 73 patients with long COVID Population: Adults ≥18 years with Long COVID recruited from a post-COVID outpatient clinic at Hospital Clinic Barcelona	Mean Age: 57.4 ± 10.5 years Gender (%): 56.2% male	OSA Definition: OSA diagnosed using full overnight in-laboratory polysomnography (PSG); OSA defined as AHI ≥5 events/h OSA Severity: Mild: AHI 5– < 15 events/h — 27.4% Moderate: AHI 15–30 events/h−24.6% Severe: AHI >30 events/h−31.5% Moderate-to-severe OSA (AHI ≥15): 56.1%
Calik and Peker (2026)	Country: Turkey Study Design: Matched case-control study	Sample Size: 304 Population: Adults aged ≥18 years evaluated in a sleep laboratory: COVID-19 history group (*n* = 152) and matched controls without COVID-19 history (*n* = 152). Groups were individually matched for age (±3 years), sex, and BMI (±3 kg/m^2^).	Mean Age: COVID-19 group: 53.7 ± 12.4 years; Control group: 53.2 ± 12.6 years Gender (%): Male: 75.7% in both groups; Female: 24.3% in both groups	OSA Definition: OSA defined by overnight polysomnography as AHI ≥15 events/h. REM-predominant OSA defined as AHI ≥15 events/h and REM-AHI/non-REM-AHI ≥2. OSA Severity: AHI ≥5: 84.9% in COVID-19 group vs. 100.0% in controls; AHI ≥15: 50.0% vs. 77.6%. REM-predominant OSA: 47.6% vs 25.7%. No separate mild/moderate/severe OSA classification reported.
Celik et al. (2026)	Country: Turkey Study Design: Case–control study	Sample Size: 120 Population: Adults with a history of COVID-19 and matched non-COVID controls.	Mean Age: 55 ± 11.8 years Gender (%): Male: 78.3%; Female: 21.7%	OSA Definition: AHI ≥ 5 events/hour OSA Severity: Not reported
Changela et al. (2026)	Country: USA Study Design: Retrospective cohort study	Sample Size: 910393 Population: Eligible patients with COVID-19 and COVID-negative controls; COVID+ patients were classified as hospitalized or non-hospitalized.	Mean Age: NR Gender (%): NR	OSA Definition: New-onset obstructive sleep apnea (OSA) identified after COVID-19; study did not define/diagnose Long COVID/PASC. OSA Severity: NR

**Table 3b:** Study Characteristics.

Study	Long COVID definition	Follow-up duration	Mechanisms studied	Outcomes measured
Jarosch et al. (2024)	Post-COVID-19 Syndrome defined according to WHO criteria: symptoms ≥3 months after COVID-19 onset, lasting ≥2 months, not explained by another diagnosis.	Single-night PSG assessment; mean time since COVID-19 infection 8.5 ± 4.5 months.	No mechanistic analysis, objective sleep parameters assessed (AHI, sleep architecture, oxygenation, PLM).	Primary outcome: AHI (Apnea–Hypopnea Index). Secondary outcomes: PLM index, sleep efficiency, SpO_2_ parameters, PSQI score, lung function, and 1-min sit-to-stand test.
Quan et al. (2024)	PASC defined as ≥3 symptoms persisting >3 months after COVID-19 infection.	No follow-up (cross-sectional study)	No biological mechanisms were evaluated; findings are based on epidemiological association.	Individual PASC symptoms and overall PASC (≥3 symptoms >3 months); adjusted odds ratios.
Labarca et al. (2022)	Not formally defined; based on follow-up at 4 months and 1 year after confirmed infection	Two follow-ups at 4 months and 12 months	Sleep-disordered breathing, inflammatory, cardiometabolic, pulmonary, neurocognitive	Sleep metrics, inflammatory markers, metabolic labs, pulmonary function, mental/cognitive outcomes
Coelho et al. (2024)	Post-acute sequelae ≥30 days after COVID diagnosis	Not clearly specified	Association between COVID-19 and new sleep disorder diagnoses	Type and frequency of sleep disorder syndromes diagnosed in PASC patients
Zhou et al. (2024)	Based on definitions used in included studies	Varied across included studies	Association between pre-existing sleep disturbances and risk/severity of COVID-19 infection	Risk of COVID-19 infection, severity, hospitalization, mortality
Mandel et al. (2023)	Long Covid definition: Post-Acute Sequelae of SARS-CoV-2 infection (PASC) refers to ongoing, relapsing, or new symptoms, or other health effects occurring after the acute phase of SARS-CoV-2 infection	N3C 100-190 PCORnet 30-180 PEDSnet 28-179	Association between pre-existing OSA and risk of developing PASC	Post-acute sequelae of SARS-CoV-2 infection (PASC) identified during the post-acute period and adjusted hazard ratios (aHRs) across EHR networks
Ljunggren et al. (2026)	NR/Not studied. The study investigated COVID-19 severity, not Long COVID	COVID-19 observation period: Jan 2020–May 2022. PAP duration was assessed from initiation until end of 2019; PAP adherence was available from 1-year follow-up data in a subsample	RAAS upregulation, ACE2-related pathways, systemic inflammation, and intermittent hypoxia were discussed as possible mechanisms. The study specifically assessed the relationship between ODI/intermittent hypoxia and COVID-19 severity	COVID-19 severity: mild (non-hospitalized), severe (hospitalized), and critical (ICU admission or death)
Mery et al. (2026)	Symptoms persisting ≥12 weeks after acute COVID-19, not explained by another diagnosis, according to WHO and NASEM definitions.	Patients were assessed 1.8 ± 0.8 years after acute COVID-19.	No specific biological mechanism was directly tested; sleep disruption and intermittent hypoxemia were discussed as possible mechanisms.	OSA prevalence/severity; AHI; ODI; sleep quality (PSQI); fatigue (FSS); dyspnea (mMRC); daytime sleepiness (ESS); insomnia (ISI); anxiety/depression (HADS); lung function, and long COVID symptoms.
Calik and Peker (2026)	Long-COVID was not diagnosed using a formal clinical definition. Fatigue reported as ‘frequent/very frequent' was used as a surrogate marker of long-COVID.	PSG was performed within 24 months after COVID-19 infection; among participants with verifiable documentation (n=76), mean interval was 14.1 ± 8.6 months (range 0–24 months).	No biological mechanism was directly tested. Possible mechanisms discussed included residual neuromuscular effects, altered arousal threshold/REM density, residual hypoxia or impaired gas exchange, and changes in sleep-stage balance.	OSA prevalence and phenotype; total/REM/non-REM AHI; REM-AHI/non-REM-AHI ratio; sleep architecture; oxygenation; sleep symptoms; ESS; and fatigue.
Celik et al. (2026)	NM	approximately one year after infection	Upper airway collapsibility; muscle compensation; ventilatory control (loop gain); arousal threshold.	PSG parameters (AHI, ODI, SpO_2_, arousal index, sleep architecture) and OSA pathophysiological traits.
Changela et al. (2026)	Not applicable; the study did not define or diagnose Long COVID/PASC. It evaluated new-onset OSA after COVID-19.	Up to 4.5 years after COVID-19	NR	New-onset OSA after COVID-19; comparison of hospitalized and non-hospitalized COVID-19 groups with COVID-negative controls.

**Table 4a:** Study findings.

Study	Clinical findings/main results	CPAP/treatment effect	Confounders/limitations	Long COVID criteria/duration	COVID hospitalization
Jarosch et al. (2024)	Main clinical findings: PCS patients showed significantly higher prevalence of sleep apnea (60% vs 10%) and higher AHI compared to controls. Main Results: SAME MAINCLINICAL FINDINGS	Treatment (CPAP): Not an interventional study; APAP therapy initiated post-diagnosis in 40% of PCS patients. Effect of Treatment: Treatment effect not evaluated; APAP was initiated but no outcome analysis reported.	Confounders: Higher BMI in PCS group (potential confounder) Limitations: Potential confounding by BMI (although not correlated with AHI); inclusion of PCS patients from pulmonary rehabilitation limiting generalizability, study design, small sample size.	Long COVID Diagnostic Criteria (WHO/NASEM/Other): WHO criteria Long COVID Symptom Duration Required: Symptoms lasting ≥2 months, occurring 3 months from the start of COVID-19.	COVID-19 Hospitalization Status: Acute hospitalization status not individually reported. PCS participants were recruited during inpatient pulmonary rehabilitation. Hospitalized Patients (%): Not reported. Non-Hospitalized Patients (%): Not reported.
Quan et al. (2024)	Main clinical findings: Pre-existing obstructive sleep apnea was independently associated with an approximately two-fold increased risk of developing PASC (long COVID), even after adjustment for demographic and clinical confounders. Main Results: SAME MAINCLINICAL FINDINGS	Treatment (CPAP): OSA treatment status (current or past treatment) was self-reported; no interventional therapy administered as part of the study. Effect of Treatment: Treated OSA was associated with attenuation of the association between OSA and PASC in multivariable logistic regression models.	Confounders: Adjusted for age, sex, BMI, number of comorbidities, vaccination status (boosted vs. not), education level, employment status, and household income. Limitations: Self-reported OSA and COVID status; cross-sectional design; potential recall bias; lack of objective sleep testing (no PSG/AHI data).	Long COVID Diagnostic Criteria (WHO/NASEM/Other): No formal WHO/NASEM definition; symptom-based PASC models Long COVID Symptom Duration Required: ≥3 months after COVID-19 infection	COVID-19 Hospitalization Status: Hospitalization status not reported for the COVID-positive cohort Hospitalized Patients (%): Not reported. Non-Hospitalized Patients (%): Not reported.
Labarca et al. (2022)	Main clinical findings: OSA was associated with higher risk of glycemic abnormalities, pulmonary impairment, neurocognitive decline, and increased inflammatory burden at 1 year after COVID-19. Main Results: SAME MAINCLINICAL FINDINGS	Treatment (CPAP): NOT mentioned Effect of Treatment: NOT mentioned	Confounders: age, gender, BMI, and development of ARDS during the acute infection Limitations: Small sample, observational design, residual confounding, HSAT diagnosis, single-region cohort	Long COVID Diagnostic Criteria (WHO/NASEM/Other): Not formally defined as WHO/NASEM Long COVID Long COVID Symptom Duration Required: Follow-up at 4 months and 1 year; no formal minimum Long COVID duration specified	COVID-19 Hospitalization Status: COVID-19 survivors; hospitalization percentage not separately reported Hospitalized Patients (%): Not reported. Non-Hospitalized Patients (%): Not reported.
Coelho et al. (2024)	Main clinical findings: Increased risk of new sleep disorders after COVID-19 infection Main Results: Significantly higher hazard ratio for sleep disorders post-COVID	Treatment (CPAP): Not evaluated Effect of Treatment: Not evaluated	Confounders: Individual background, comorbidities, obesity, viral infection, use of medications. Limitations: The relatively small number of cases and initial screening introduce bias	Long COVID Diagnostic Criteria (WHO/NASEM/Other): PASC/Long COVID; no formal WHO/NASEM diagnostic criterion reported Long COVID Symptom Duration Required: Not reported.	COVID-19 Hospitalization Status: Not reported. Hospitalized Patients (%): Not reported. Non-Hospitalized Patients (%): Not reported.
Zhou et al. (2024)	Main clinical findings: Pre-existing sleep disturbances associated with increased risk of COVID-19 and worse outcomes Main Results: Significant pooled association between sleep disturbances and higher COVID-19 risk/severity	Treatment (CPAP): Not evaluated Effect of Treatment: Not evaluated	Confounders: age, income level, male proportion, exposure measurement, outcome measurement, study design, OR, literature quality. Limitations: Heterogeneity between studies, observational design, variable definitions, possible publication bias	Long COVID Diagnostic Criteria (WHO/NASEM/Other): Heterogeneous across included studies; included studies used varying Long COVID definitions ≥1 month vs ≥3 months Long COVID Symptom Duration Required: Varied across included studies: ≥1 month or ≥3 months.	COVID-19 Hospitalization Status: NR for the Long COVID population as a baseline characteristic. Hospitalized Patients (%): Not reported. Non-Hospitalized Patients (%): Not reported.
Mandel et al. (2023)	Main clinical findings: Pre-existing OSA was associated with an increased risk of developing post-acute sequelae following SARS-CoV-2 infection Main Results: Patients with pre-existing OSA had significantly higher adjusted hazard ratios for PASC compared to those without OSA	Treatment (CPAP): Not evaluated Effect of Treatment: Not evaluated	Confounders: Adjusted for age, sex, race, ethnicity, hospitalization status, obesity,comorbidities. Limitations: incomplete covid documentation, no vaccination adjustment, no OSA severity data	Long COVID Diagnostic Criteria (WHO/NASEM/Other): Network-specific PASC computable phenotypes; not a single WHO/NASEM definition. Long COVID Symptom Duration Required: Not reported.	COVID-19 Hospitalization Status: Reported; categorized as not hospitalized, hospitalized, or hospitalized with ICU-level care. Hospitalized Patients (%): N3C 8.37%; PCORnet 17.70%; PEDSnet 4.71% Non-Hospitalized Patients (%): N3C 91.63%; PCORnet 82.30%; PEDSnet 95.29%
Ljunggren et al. (2026)	Main clinical findings: Moderate-to-severe OSA was associated with increased risk of severe and critical COVID-19. Higher intermittent hypoxia (ODI) was associated with greater COVID-19 severity. Main Results: Severe COVID: 8.9% vs 3.6%; critical COVID: 2.6% vs 1.3% in patients with vs without OSA. Longer PAP duration was associated with lower risk of critical COVID.	Treatment (CPAP): PAP treatment; adherence defined as ≥4 h/night. Most registered OSA patients were PAP-treated Effect of Treatment: OSA remained associated with severe/critical COVID-19 in both PAP-adherent and non-adherent patients. However, longer PAP duration was associated with lower risk of critical COVID-19.	Confounders: Age, sex, education, diabetes, hypertension, ischemic heart disease, heart failure, regular inhaler use, severe respiratory disease, severe chronic kidney disease, healthcare region; BMI was additionally included in OSA-severity analyses Limitations: BMI data were unavailable for the full population, and some undiagnosed OSA may have been present in the comparison group, potentially underestimating the OSA effect.	Long COVID Diagnostic Criteria (WHO/NASEM/Other): Not applicable; study assessed acute COVID-19 severity rather than Long COVID/PASC. Long COVID Symptom Duration Required: Not applicable	COVID-19 Hospitalization Status: Mild = non-hospitalized; severe = hospitalized but not ICU/death; critical = ICU admission or death. Hospitalized Patients (%): 4.9% hospitalized (3.6% severe + 1.3% critical) in general COVID population Non-Hospitalized Patients (%): 95.1% non-hospitalized in general COVID population
Mery et al. (2026)	Main clinical findings: OSA was highly prevalent in Long COVID patients (83.6%), with 56.1% having moderate-to-severe OSA. OSA severity was not significantly associated with most Long COVID symptoms, but higher AHI was associated with insomnia. Clinically relevant OSA was also associated with a higher prevalence of coexisting insomnia and OSA. Main Results: SAME MAINCLINICAL FINDINGS	Treatment (CPAP): Patients with clinically relevant OSA were treated according to international recommendations. CPAP was prescribed mainly for severe OSA. Effect of Treatment: Treatment effect was not formally evaluated; only anecdotal improvement in long COVID symptoms was reported.	Confounders: Age, BMI, and hospitalization status were considered in adjusted analyses. Limitations: Small sample size, single-center observational design, possible selection bias and possible undiagnosed pre-existing OSA.	Long COVID Diagnostic Criteria (WHO/NASEM/Other): WHO and National Academies of Sciences, Engineering, and Medicine (NASEM) definitions. Long COVID Symptom Duration Required: Symptoms present 12 weeks after acute COVID-19 infection.	COVID-19 Hospitalization Status: LC outpatient cohort; most had previously been hospitalized for COVID-19 pneumonia. Hospitalized Patients (%): 73.9% (54/73) Non-Hospitalized Patients (%): 26.0% (19/73)
Calik and Peker (2026)	Main clinical findings: Post-COVID participants had lower overall OSA prevalence but a significantly higher prevalence of REM-predominant OSA. Fatigue was more frequent among participants with REM-predominant OSA. Main Results: Prior COVID-19 infection was independently associated with REM-predominant OSA (OR 3.144, 95% CI 1.885–5.245, p < 0.001) after adjustment for age, sex, BMI, and smoking. REM-predominant OSA was independently associated with fatigue (OR 2.183, 95% CI 1.290–3.693, p=0.004).	Treatment (CPAP): Not evaluated; this was an observational case-control study. Effect of Treatment: Not evaluated; no treatment intervention was studied.	Confounders: Age, sex, BMI, and current smoking status. Limitations: Single tertiary referral center; retrospective design; referral and recall/reporting bias; detailed hospitalization/ICU/ventilatory-support data unavailable for most COVID-19 cases; no long-term follow-up; case-control design cannot establish causality.	Long COVID Diagnostic Criteria (WHO/NASEM/Other): No formal WHO/NASEM/other diagnostic criteria. Fatigue reported as “frequent/very frequent” was used as a surrogate marker of long-COVID. Long COVID Symptom Duration Required: Not reported; no minimum symptom duration specified.	COVID-19 Hospitalization Status: Not reported. Hospitalization status during acute COVID-19 was unavailable for the majority of cases. Hospitalized Patients (%): Not reported. Non-Hospitalized Patients (%): Not reported.
Celik et al. (2026)	Main clinical findings: No significant differences in the four primary OSA pathophysiological traits (upper airway collapsibility, loop gain, arousal threshold, and muscle compensation) between participants with and without prior COVID-19 after adjustment. Ventilatory response to arousal (VRA) was significantly higher in the COVID-19 group. Main Results: No significant differences in primary OSA physiological traits; significantly higher ventilatory response to arousal (VRA) in the post-COVID group (adjusted difference 6.6% eupnea, p=0.022).	Treatment (CPAP): NOT mentioned Effect of Treatment: NOT mentioned	Confounders: Age, gender, BMI, and AHI. Limitations: Control group had more severe OSA; no pre-infection PSG data; PSG performed ~1 year after COVID-19; physiological traits were estimated rather than measured using gold-standard invasive methods; predominantly male sample (78%), limiting generalizability.	Long COVID Diagnostic Criteria (WHO/NASEM/Other): Not applicable. Long COVID Symptom Duration Required: Not applicable.	COVID-19 Hospitalization Status: Not reported. Hospitalized Patients (%): Not reported. Non-Hospitalized Patients (%): Not reported.
Changela et al. (2026)	Main clinical findings: New-onset OSA risk was higher after COVID-19 in both hospitalized and non-hospitalized patients. Main Results: Hospitalized COVID+ vs COVID–: HR 1.41; non-hospitalized COVID+ vs COVID–: HR 1.33.	Treatment (CPAP): Not evaluated Effect of Treatment: Not evaluaterd	Confounders: NR Limitations: Large observational database study; residual confounding and observational design limitations.	Long COVID Diagnostic Criteria (WHO/NASEM/Other): Not applicable; no formal WHO/NASEM Long COVID/PASC definition was used. Long COVID Symptom Duration Required: Not applicable/no Long COVID symptom-duration criterion reported.	COVID-19 Hospitalization Status: COVID+ patients were explicitly divided into hospitalized and non-hospitalized groups. Hospitalized Patients (%): 25.3 Non-Hospitalized Patients (%): 74.7

**Table 4b:** Study findings.

Study	COVID Severity/Clinical Support	OSA Diagnostic Criteria/Severity Distribution	OSA vs Non-OSA/Cognitive Findings	Additional OSA Burden
Jarosch et al. (2024)	COVID-19 Severity: Not reported. ICU Admission (%): Not reported. Mechanical Ventilation/Intubation (%): Not reported. Oxygen Requirement During Acute COVID-19 (%): Not reported.	OSA Definition & Diagnostic Criteria: Sleep apnea assessed by PSG using AHI. Mild 5–14, moderate 15–29, severe >30 events/h. OSA Severity Distribution: Mild: 9 (45%); Moderate: 1 (5%); Severe: 2 (10%). OSA-specific severity not reported.	OSA vs. Non-OSA Comparison & Symptom Burden: Not assessed; comparison was PCS vs. healthy controls, not PCS with vs. without OSA. Cognitive Assessment & Findings: Not reported.	Evidence for Additional OSA Burden: Not assessed.
Quan et al. (2024)	COVID-19 Severity: Not reported. ICU Admission (%): Not reported. Mechanical Ventilation/Intubation (%): Not reported. Oxygen Requirement During Acute COVID-19 (%): Not reported.	OSA Definition & Diagnostic Criteria: Self-reported current OSA diagnosis (treated or untreated) or ≥2 OSA symptoms (snoring, witnessed apnea, and/or sleepiness). Study 5 OSA Severity Distribution: Not reported.	OSA vs. Non-OSA Comparison & Symptom Burden: OSA was associated with a greater likelihood of all PASC-related physical, cognitive, and mental health symptoms compared with non-OSA. Cognitive Assessment & Findings: Self-reported cognitive symptoms; OSA was associated with significantly higher odds of all assessed cognitive symptoms.	Evidence for Additional OSA Burden: OSA was strongly associated with increased PASC risk and persistent physical, cognitive, and mental health symptoms; untreated OSA was identified as an important risk factor.
Labarca et al. (2022)	COVID-19 Severity: Mild 30.0%; Moderate 28.3%; Severe 41.6% ICU Admission (%): 56.6% (34/60) Mechanical Ventilation/Intubation (%): Not reported. Oxygen Requirement During Acute COVID-19 (%): Not reported.	OSA Definition & Diagnostic Criteria: OSA defined as RDI ≥5 events/h on a home sleep apnea test (HSAT); moderate-to-severe OSA defined as RDI ≥15 events/h. OSA Severity Distribution: Mild OSA: 22/37 (59.4%); moderate OSA: 12/37 (32.4%); severe OSA: 3/37 (8.1%).	OSA vs Non-OSA Comparison & Symptom Burden: OSA patients had greater long-term metabolic, pulmonary, and neurocognitive burden than non-OSA patients, including higher insulin resistance and persistent abnormal DLCO; no significant differences in sleep or mental health symptoms at 1 year. Cognitive Assessment & Findings: MoCA at 1 year (< 25 = neurocognitive impairment); OSA patients had significantly lower MoCA scores than non-OSA patients (22 vs 25, p=0.02).	Evidence for Additional OSA Burden: OSA was associated with greater metabolic, pulmonary, and neurocognitive sequelae up to 1 year after COVID-19.
Coelho et al. (2024)	COVID-19 Severity: Not reported. ICU Admission (%): Not reported. Mechanical Ventilation/Intubation (%): Not reported. Oxygen Requirement During Acute COVID-19 (%): Not reported.	OSA Definition & Diagnostic Criteria: OSA diagnosed using ICSD-3-R criteria; all patients underwent PSG. OSA Severity Distribution: Not reported.	OSA vs Non-OSA Comparison & Symptom Burden: Not assessed. Cognitive Assessment & Findings: Not reported.	Evidence for Additional OSA Burden: Not assessed.
Zhou et al. (2024)	COVID-19 Severity: NR as a baseline severity classification for the Long COVID population. ICU Admission (%): Not reported. Mechanical Ventilation/Intubation (%): Not reported. Oxygen Requirement During Acute COVID-19 (%): Not reported.	OSA Definition & Diagnostic Criteria: OSA definition varied across studies; no single AHI-based definition was used. OSA Severity Distribution: Not reported.	OSA vs Non-OSA Comparison & Symptom Burden: OSA was associated with Long COVID risk, but symptom burden in Long COVID patients with vs without OSA was not assessed. Cognitive Assessment & Findings: Not reported.	Evidence for Additional OSA Burden: Not assessed.
Mandel et al. (2023)	COVID-19 Severity: Hospitalized without severe illness vs. hospitalized with ICU, ventilation, or vasopressors; exact severity categories beyond these were not separately reported ICU Admission (%): N3C 0.99%; PCORnet 4.75%; PEDSnet 0.89% (hospitalized with ICU-level care) Mechanical Ventilation/Intubation (%): Not separately reported; ventilation was included within the severe hospitalization category Oxygen Requirement During Acute COVID-19 (%): Not reported.	OSA Definition & Diagnostic Criteria: Pre-existing OSA defined as ≥2 records of OSA-related ICD-9-CM/ICD-10-CM or equivalent SNOMED-CT diagnostic codes on separate days. OSA Severity Distribution: Not reported.	OSA vs. Non-OSA Comparison & Symptom Burden: Pre-existing OSA was associated with higher odds of probable PASC in adults; individual symptom burden was not assessed. Cognitive Assessment & Findings: Not assessed.	Evidence for Additional OSA Burden: Pre-existing OSA was associated with increased odds of probable PASC in adults after adjustment.
Ljunggren et al. (2026)	COVID-19 Severity: General population: mild 95.1%, severe 3.6%, critical 1.3%. Registered OSA: mild 88.5%, severe 8.9%, critical 2.6%. ICU Admission (%): Not separately reported; critical was 1.3% overall and 2.6% in registered OSA, including ICU admission or death. Mechanical Ventilation/Intubation (%): Not reported. Oxygen Requirement During Acute COVID-19 (%): Not reported.	OSA Definition & Diagnostic Criteria: OSA identified from the Swedish National Register of Respiratory Failure (Swedevox) among patients who started PAP treatment for OSA during 2015–2019. OSA Severity Distribution: OSA severity was assessed using the apnea-hypopnea index (AHI) and oxygen desaturation index (ODI), categorized as mild (< 15 events/h), moderate (15- < 30 events/h), and severe (≥30 events/h).	OSA vs. Non-OSA Comparison & Symptom Burden: Symptom burden was not assessed. Cognitive Assessment & Findings: Not assessed.	Evidence for Additional OSA Burden: Not assessed.
Mery et al. (2026)	COVID-19 Severity: Initial COVID-19 severity was represented using hospitalization as a surrogate severity marker; detailed ICU/ventilation/oxygen percentages were not reported. ICU Admission (%): Not reported. Mechanical Ventilation/Intubation (%): Not reported. Oxygen Requirement During Acute COVID-19 (%): Not reported.	OSA Definition & Diagnostic Criteria: OSA was diagnosed by full overnight polysomnography (PSG). OSA was defined as AHI ≥5 events/h. Clinically relevant OSA was defined as AHI ≥15/h. OSA Severity Distribution: Among the 73 LC patients, OSA was mild in 27.4% (AHI 5– < 15/h), moderate in 24.6% (AHI 15–30/h), and severe in 31.5% (AHI >30/h).	OSA vs. Non-OSA Comparison & Symptom Burden: Patients with moderate–severe OSA (AHI ≥15/h) were older, more often male, and had higher BMI than those with no/mild OSA. The overall prevalence of LC symptoms was not significantly different, but the symptom burden was higher in patients with no/mild OSA. Cognitive Assessment & Findings: Self-reported cognitive complaints were assessed using validated questionnaires; no objective cognitive testing was reported.	Evidence for Additional OSA Burden: Insomnia was the only LC symptom significantly associated with OSA severity. AHI showed a mild positive correlation with ISI (r = 0.30, p = 0.014), and after adjustment, AHI increased by 0.8 for each 1-point increase in ISI.
Calik and Peker (2026)	COVID-19 Severity: Not reported/could not be reliably analyzed. ICU Admission (%): Not reported. Mechanical Ventilation/Intubation (%): Not reported. Oxygen Requirement During Acute COVID-19 (%): Not reported.	OSA Definition & Diagnostic Criteria: OSA was diagnosed by full overnight polysomnography (PSG). OSA was defined as AHI ≥15/h. OSA Severity Distribution: The study used AHI ≥15/h as the OSA threshold and focused on the REM-predominant phenotype.	OSA vs. Non-OSA Comparison & Symptom Burden: The study compared REM-predominant OSA vs non-REM-predominant OSA. REM-predominant OSA had less snoring, witnessed apneas, nocturia, and excessive daytime sleepiness, but more fatigue. Overall, classical OSA symptoms were also less frequent in the post-COVID group despite PSG-confirmed OSA. Cognitive Assessment & Findings: Not assessed.	Evidence for Additional OSA Burden: Fatigue was the main additional burden associated with REM-predominant OSA.
Celik et al. (2026)	COVID-19 Severity: Not reported. ICU Admission (%): Not reported. Mechanical Ventilation/Intubation (%): Not reported. Oxygen Requirement During Acute COVID-19 (%): Not reported.	OSA Definition & Diagnostic Criteria: OSA defined as AHI ≥5 events/hour on overnight polysomnography (PSG). Apnea was defined as ≥90% cessation of airflow; hypopnea as ≥30% reduction in nasal pressure and/or thoraco-abdominal movement for ≥10 s, accompanied by ≥3% oxygen desaturation or an arousal, according to AASM criteria. OSA Severity Distribution: Not reported.	OSA vs. Non-OSA Comparison & Symptom Burden: Not assessed. Cognitive Assessment & Findings: Not assessed.	Evidence for Additional OSA Burden: Not assessed.
Changela et al. (2026)	COVID-19 Severity: Hospitalized vs non-hospitalized COVID-19 infection; no further acute severity categories reported. ICU Admission (%): Not reported. Mechanical Ventilation/Intubation (%): Not reported. Oxygen Requirement During Acute COVID-19 (%): Not reported.	OSA Definition & Diagnostic Criteria: OSA was defined as a new ICD-10 diagnosis of OSA after the COVID-19 index date. No objective diagnostic criteria (AHI/ODI/PSG) were reported. OSA Severity Distribution: Not reported.	OSA vs. Non-OSA Comparison & Symptom Burden: Symptom burden was not assessed. Cognitive Assessment & Findings: Not assessed.	Evidence for Additional OSA Burden: Not assessed.

## 3 Results

A total of 184 records were identified through database searching covering the period 2020–2026, comprising 179 records from the original search and five additional records identified through a subsequent search update. After removing 49 duplicates, 135 records remained for screening. Of these, 121 records were excluded. The full texts of 14 studies were assessed for eligibility, of which 3 were excluded. Finally, 11 studies were included in this review (Figure 1).

**Figure 1:**
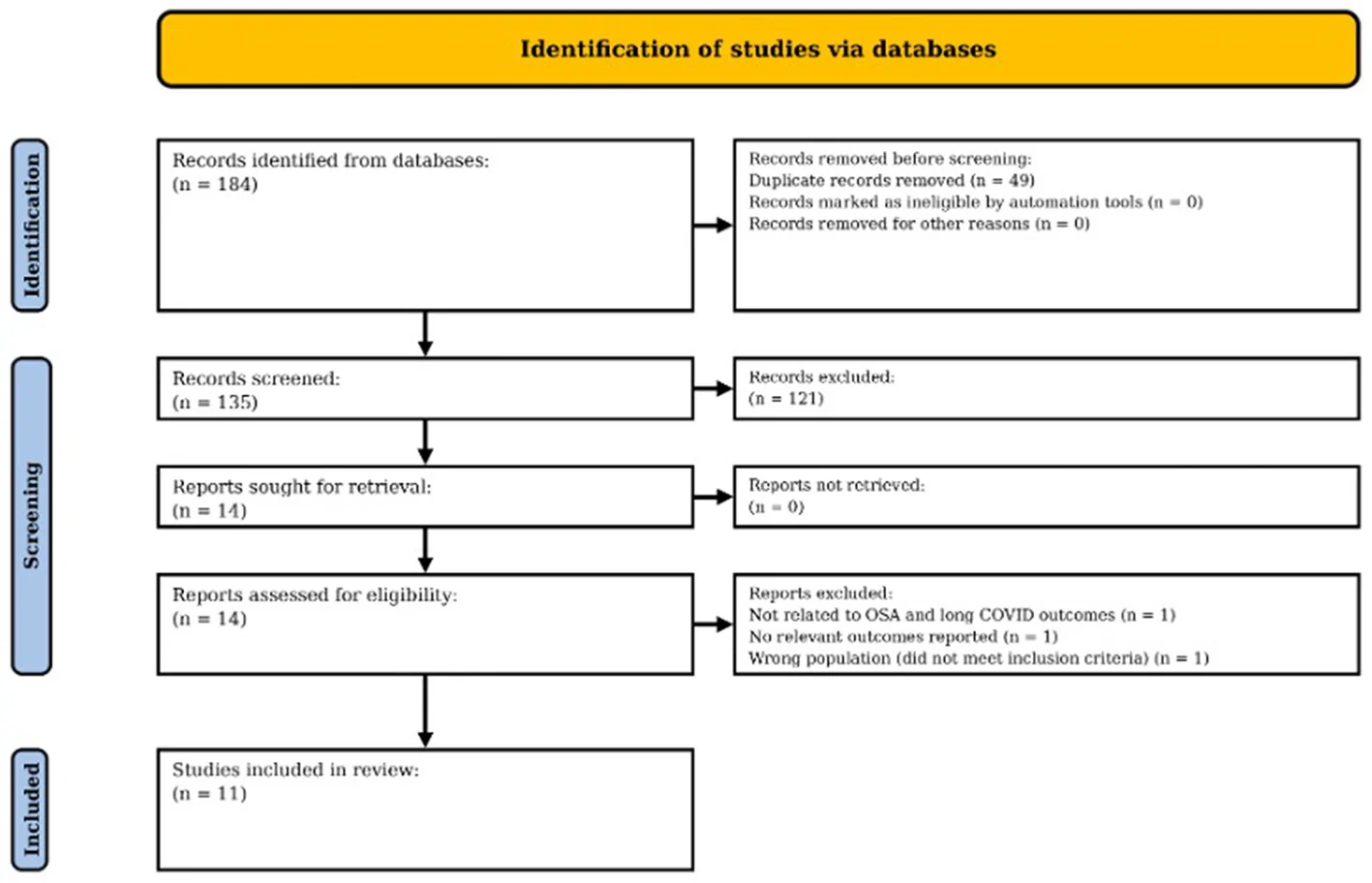
PRISMA flow diagram of study selection.

The included studies were conducted in multiple countries, including the United States, Germany, Chile, Sweden, Spain, and Turkey, with several studies utilizing data from multiple countries or large national registries. The study populations included adults with confirmed COVID-19 infection, individuals with post-COVID-19 syndrome, and general population samples. Some studies included comparative groups, comprising individuals with and without obstructive sleep apnea (OSA), as well as COVID-19 positive and negative populations. One study included both adult and pediatric populations using separate databases.

Across studies, different study designs were used including cross-sectional studies, cohort studies, retrospective/longitudinal cohort studies, a case-control polysomnographic study, a physiological trait analysis, meta-analyses, and EHR-based studies. Different sample sizes were utilized in various studies ranging from a relatively smaller sample size of less than 50 subjects to larger population-based studies involving hundreds of thousands or even nearly a million subjects. The selected literature examined different types and directions of associations between OSA and COVID-19-related outcomes, including Long COVID or PASC. Additional objectives included describing related clinical outcomes and identifying potential risk factors. The characteristics of the included studies are summarized in Tables 3a, b. Long COVID definitions varied across the included studies. Some studies applied WHO or NASEM criteria, or referenced both frameworks, whereas others used study-specific definitions or did not report a formal Long COVID definition. Required symptom duration also varied or was not reported. Acute COVID-19 hospitalization status and severity were variably reported across studies, with some studies distinguishing hospitalized from non-hospitalized patients or reporting severity categories. Data on ICU admission, mechanical ventilation/intubation, and oxygen requirements during acute COVID-19 were less consistently available and were not reported in several studies. Detailed study-level data are presented in Tables 3a, b, 4a, b.

### 3.1 Association between OSA and long COVID

The included studies examined different types and directions of associations between OSA and COVID-19-related outcomes, including Long COVID/PASC. Studies examining pre-existing OSA in relation to subsequent PASC or post-COVID outcomes were distinguished from studies identifying OSA or sleep abnormalities within Long COVID populations. Other studies examined COVID-19 in relation to subsequent new-onset OSA or OSA-related physiological characteristics, while one study examined pre-existing OSA in relation to acute COVID-19 severity. The systematic review and meta-analysis were considered separately as secondary evidence to avoid treating overlapping evidence as an independent finding. However, the evidence varied in design, OSA ascertainment, PASC definitions, and adjustment for potential confounders, limiting direct comparison across studies.

### 3.2 Pathophysiological mechanisms

Five of the included studies discussed or proposed mechanisms that could link OSA with Long COVID. Intermittent hypoxia and oxygen desaturation were frequently identified as potential biological pathways, together with systemic inflammation and sleep disruption. A more recent polysomnographic trait analysis directly tested several of these proposed mechanisms and found no meaningful differences in upper airway collapsibility, loop gain, or arousal threshold between individuals with and without a history of COVID-19, but did identify a greater ventilatory response to arousal among those with prior COVID-19, suggesting a narrower physiological signature than earlier mechanistic hypotheses implied. These mechanisms should therefore continue to be interpreted as hypothesized explanations rather than established causal pathways because objective mechanistic measures remain limited across the included studies. Sleep fragmentation and circadian disturbances were also observed in patients with post-COVID conditions. Figure 2 illustrates the hypothesized relationship between OSA and Long COVID syndrome through shared pathophysiological mechanisms.

**Figure 2:**
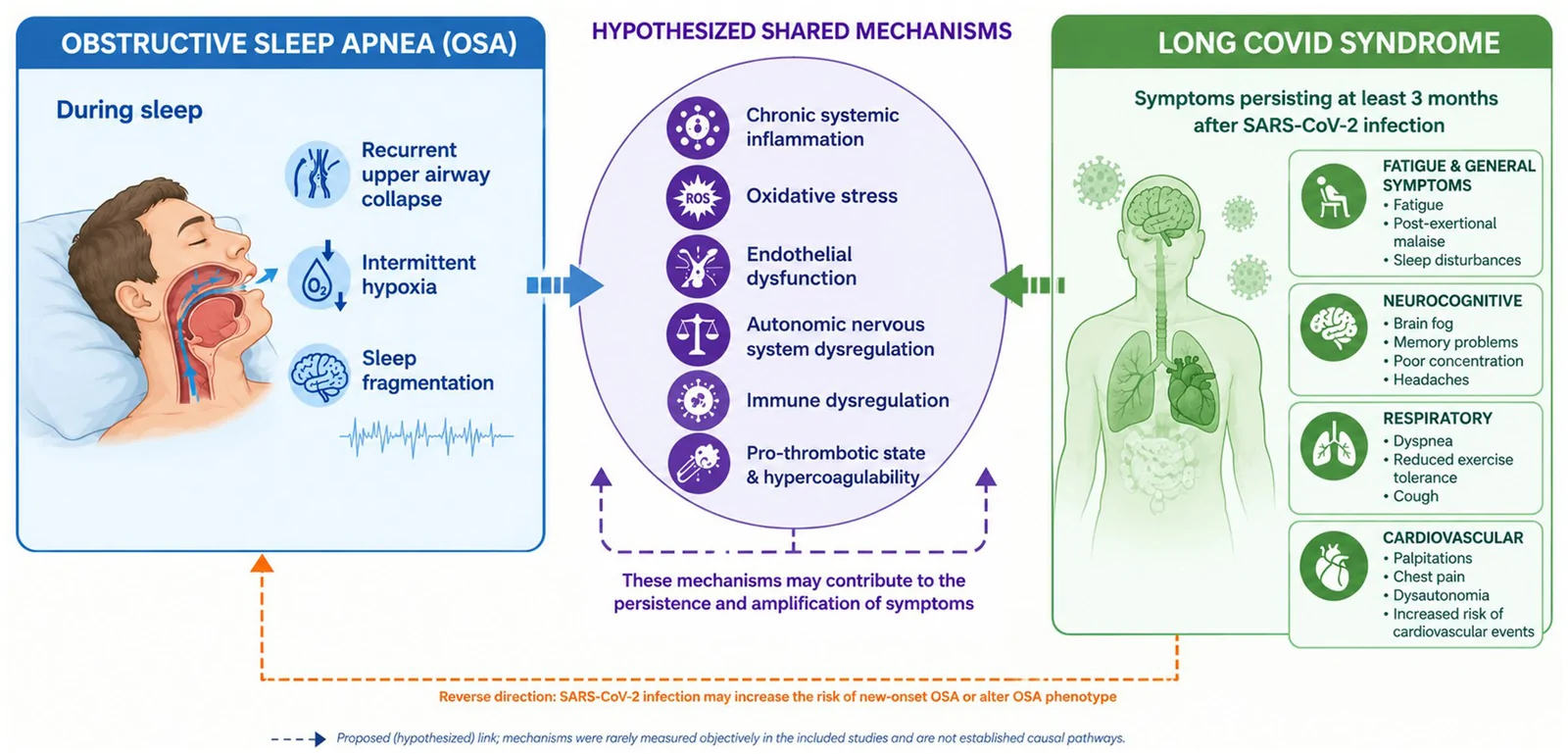
Hypothesized relationships between obstructive sleep apnea (OSA) and Long COVID.

### 3.3 Clinical outcomes

Seven of the included studies reported clinical manifestations or outcomes across the different OSA–COVID-19 relationships examined. Reported outcomes included pulmonary impairment, neurocognitive symptoms, metabolic abnormalities, fatigue, insomnia, hypersomnolence, circadian disturbances, and, in more recently identified studies, a REM-predominant OSA phenotype associated with fatigue and an elevated risk of pulmonary hypertension and heart failure among patients who developed new-onset OSA after COVID-19. Because outcome definitions and assessment methods differed across studies, direct comparisons between Long-COVID patients with and without OSA were limited, with inconsistent findings regarding additional neurocognitive, pulmonary, and overall symptom burden.The findings were mapped descriptively rather than quantitatively pooled.

### 3.4 Severity and persistence

Four of the included studies examined severity or persistence across different OSA–COVID-19 relationships. These included studies evaluating the persistence of post-COVID outcomes in individuals with OSA, as well as a large retrospective cohort demonstrating elevated risk of new-onset OSA for up to 4.5 years after COVID-19 infection. Long-term follow-up data showed that pulmonary abnormalities and neurocognitive impairment persisted up to 1 year after infection in patients with OSA. Sleep disturbances were associated with a higher risk of prolonged symptoms.

### 3.5 Effect of treatment

Evidence regarding the effect of OSA treatment on Long COVID outcomes remains limited. Three included studies provided information relevant to OSA treatment. However, one of these studies evaluated positive airway pressure therapy in relation to acute COVID-19 severity rather than Long COVID outcomes. Treatment exposure, adherence, and Long COVID outcomes were not assessed consistently across studies. Therefore, the available evidence is insufficient to determine whether effective OSA treatment modifies the course of Long COVID. Furthermore, attenuation of the association between OSA and long COVID was suggested in some analyses, although the results varied among studies.

## 4 Discussion

This scoping review identified heterogeneous relationships between obstructive sleep apnea (OSA) and Long COVID/PASC-related outcomes across the included studies. Some studies examined pre-existing OSA as a potential risk factor for PASC, whereas others evaluated OSA among patients with Long COVID or examined the development of OSA following COVID-19.

Studies identified through the updated 2020–2026 search extended these findings in several ways. A prospective study of patients attending a dedicated Long-COVID clinic found a high prevalence of polysomnography-confirmed OSA, including among patients without daytime sleepiness (Mery et al., 2026), reinforcing the case for proactive OSA screening in post-COVID populations. Calik and Peker's matched case-control polysomnographic study further showed that, although overall OSA prevalence was lower among individuals with a history of COVID-19, a REM-predominant OSA phenotype was significantly more common in this group and was associated with fatigue, a core Long-COVID symptom (Calik and Peker, 2026), suggesting that COVID-19 may selectively affect upper airway collapsibility during REM sleep rather than uniformly increasing OSA burden. A nationwide register-based cohort study found that moderate-to-severe OSA independently increased the risk of severe acute COVID-19 (Ljunggren et al., 2026), while a large retrospective cohort of more than 900,000 adults found that both hospitalized and non-hospitalized COVID-19 patients had a significantly higher risk of developing new-onset OSA for up to 4.5 years after infection (Changela et al., 2026). These findings represent different directions of association and should be interpreted separately from evidence examining pre-existing OSA as a potential risk factor for Long COVID. By contrast, a detailed polysomnographic trait analysis found no meaningful differences in upper airway collapsibility, loop gain, or arousal threshold by COVID-19 history, apart from a greater ventilatory response to arousal among those with prior infection (Celik et al., 2026), indicating that the physiological signature of any COVID-19-related change in OSA pathophysiology may be narrower than earlier mechanistic hypotheses suggested.

Potential mechanisms underlying the relationship between OSA and post-COVID outcomes may include the underlying physiological burden associated with OSA. Chronic intermittent hypoxia, sleep fragmentation, and oxygen desaturation, well-established drivers of oxidative stress and systemic inflammation in OSA (Dempsey et al., 2010), may contribute to impaired recovery following COVID-19 infection (Labarca et al., 2022; Jarosch et al., 2024). In addition, inflammatory processes and sleep-related disturbances reported across studies may increase vulnerability to persistent respiratory and neurocognitive symptoms (Labarca et al., 2022; Quan et al., 2024).

Additional mechanisms may further explain the relationship between OSA and long COVID. Intermittent hypoxia and oxygen desaturation are characteristic features of OSA and may promote inflammatory responses and immune dysregulation (Labarca et al., 2022). Furthermore, sleep disruption and circadian disturbances may negatively affect physiological recovery and contribute to the persistence of post-COVID symptoms (Zhou et al., 2024; Quan et al., 2024). In people with OSA, inflammatory dysregulation may also be a significant factor in the maintenance of prolonged COVID symptoms. After recovering from an acute infection, repeated hypoxic episodes may increase oxidative stress and inflammatory activity, which may contribute to persistent respiratory and neurocognitive impairment (Labarca et al., 2022; Quan et al., 2024).

Evidence from studies specifically examining pre-existing OSA suggested a possible association with more persistent post-COVID manifestations (Zhou et al., 2024; Labarca et al., 2022). However, the observational nature and heterogeneity of the available evidence limit conclusions regarding an independent effect of OSA on the development or persistence of Long COVID. Findings from studies examining OSA within post-COVID populations or the development of OSA after COVID-19 should be interpreted separately and do not establish pre-existing OSA as a risk factor for Long COVID. In addition, Long COVID definitions and required symptom duration varied across studies, including the use of WHO, NASEM, both frameworks, study-specific criteria, or no formal definition. Acute COVID-19 hospitalization and severity characteristics were also inconsistently reported, with limited reporting of ICU admission, mechanical ventilation/intubation, and oxygen requirements. These differences should be considered when interpreting and comparing findings across studies.

The association observed between OSA and persistent symptoms such as fatigue, dyspnea, and cognitive impairment Regarding the contribution of OSA to the symptom burden of Long COVID, the available evidence remains limited and heterogeneous. Labarca et al. provided the most direct comparison between participants with and without OSA following COVID-19 and included an objective cognitive assessment using the Montreal Cognitive Assessment. Participants with OSA had lower cognitive scores and a higher prevalence of impaired pulmonary function compared with those without OSA; however, the association between OSA and neurocognitive impairment was no longer statistically significant after adjustment for potential confounders. Conversely, the prospective Long-COVID study by Mery et al. found no significant differences in most symptom measures between patients with moderate-to-severe OSA and those with no or mild OSA. These findings suggest that OSA may contribute to selected pulmonary and neurocognitive outcomes, but current evidence does not establish a consistent additional overall symptom burden among patients with Long COVID. also aligns with findings reported in earlier studies on post-COVID conditions (Quan et al., 2024). Previous studies also suggested that physiological and inflammatory mechanisms may contribute to prolonged recovery and ongoing respiratory and neurocognitive symptoms after COVID-19 infection (Jarosch et al., 2024; Labarca et al., 2022).

The variability in the observed findings may be explained by differences in study methodology and patient characteristics. Variations in OSA severity, diagnostic criteria, and duration of follow-up may influence the reported association between OSA and long COVID outcomes (Jarosch et al., 2024; Zhou et al., 2024).

The association between OSA and long COVID may also be influenced by underlying comorbidities that are commonly seen in patients with OSA, including obesity, cardiovascular disease, hypertension, and metabolic dysfunction (Miller and Cappuccio, 2020). These comorbidities have independently been associated with poorer COVID-19 outcomes and may further contribute to prolonged recovery and persistent symptoms following infection. Therefore, these shared comorbidities may partly explain or modify the observed association between OSA and Long COVID, and residual confounding cannot be excluded. Similarly, a recent study by Popovici et al. (2024) found that obesity, cardiovascular disease, older age, and smoking were important predictors of severe post-COVID OSA manifestations as well as prolonged respiratory symptoms.

However, the strength of this association was heterogeneous, as was the effect of OSA treatment on recovery outcomes. Some studies indicated a possible enhancing effect of positive airway pressure therapy, while other studies reported limited or unclear benefits. These differences may be related to variations in study populations, follow-up time, and diagnostic methods.

These results suggest a potential association between obstructive sleep apnea (OSA) and persistent post-COVID symptoms and highlight the importance of considering pre-existing OSA in patients with persistent symptoms after COVID-19 infection. The overlap of inflammatory, hypoxic, and neurocognitive features among the included studies may suggest common pathways between OSA and long COVID. These findings may carry important implications for clinical practice. Individuals with persistent post-COVID symptoms may benefit from evaluation for underlying sleep disorders, particularly OSA, as early detection may improve symptom management and long-term monitoring strategies (Quan et al., 2024; Luo et al., 2024). Similar recommendations on routine screening and long-term monitoring of sleep-related disorders in post-COVID patients have also been reported in previous studies (Popovici et al., 2024; Coelho et al., 2024).

In addition, the overlap between symptoms commonly reported in OSA and long COVID, including fatigue, daytime sleepiness, impaired concentration, and sleep disturbances, may complicate clinical assessment and delay diagnosis. The persistence of these symptoms may negatively affect daily functioning, physical performance, and psychosocial wellbeing, further increasing the long-term burden among affected individuals (Labarca et al., 2022). This overlap highlights the importance of considering sleep-related disorders when evaluating patients presenting with persistent symptoms following COVID-19 infection. Increased awareness among healthcare providers may facilitate earlier identification of OSA and support more comprehensive long-term management strategies for affected individuals (Jarosch et al., 2024; Zhou et al., 2024).

Evidence gaps identified by the scoping review included a limited number of studies specifically evaluating pre-existing OSA in relation to Long COVID, heterogeneous definitions and diagnostic approaches, limited objective assessment of biological mechanisms, and sparse evidence regarding the effect of OSA treatment on post-COVID outcomes. These gaps indicate a need for prospective studies using standardized OSA and Long COVID definitions, objective sleep assessment, and clinically meaningful longitudinal outcomes.

This review presents some limitations that need to be taken into account when evaluating the results. Firstly, the number of selected studies to be analyzed, while expanded through an updated search, remained relatively small (*n* = 11). Secondly, the heterogeneity of the selected studies was very high when considering their design, study population, measures of outcome, and follow-up period. Since most of the selected studies to be analyzed were observational studies, the causality of the relationship between OSA and long COVID was hard to prove. Also, differences in defining and diagnosing OSA and long COVID may cause inconsistency in the results obtained. The lack of sufficient evidence about the effect of OSA treatment such as continuous positive airway pressure (CPAP) on long COVID outcomes is an important limitation of this study. Another limitation is the limited number of studies directly comparing Long-COVID patients with and without OSA, particularly using objective measures of cognitive function. Therefore, the extent to which OSA independently contributes to the overall symptom burden of Long COVID remains uncertain. In addition, variations in outcome reporting and symptom assessment may have reduced comparability between studies and limited the generalizability of the findings.

Well-designed longitudinal studies are needed to help determine the causal relationship between OSA and long COVID. Future studies should also use standardized diagnostic criteria and outcome measures to improve comparability across studies. Further investigation into the underlying mechanisms, particularly hypoxia and inflammation, is required. In addition, more research evaluating the effect of interventions such as CPAP therapy on long COVID outcomes is needed.

The growing evidence regarding the relationship between OSA and long COVID underscores the importance of greater attention to sleep health following COVID-19 infection. Understanding the interaction between sleep-related disorders and post-COVID syndrome may contribute to improved patient outcomes and support more targeted management approaches.

## 5 Conclusion

Overall, the available evidence suggests an association between obstructive sleep apnea and Long COVID/PASC, but the current evidence base remains limited and heterogeneous. Intermittent hypoxia, inflammatory reactions, and sleep disturbances are among the proposed mechanisms potentially linking OSA and Long COVID/PASC. However, the evidence is still limited and inconsistent, especially concerning the influence of OSA treatment on long COVID prognosis. Therefore, the available evidence supports an association between OSA and Long COVID/PASC, although the direction of this relationship remains unclear.
